# Compassion-based positive psychology group intervention (CB-PPGI) for individuals with personality disorders awaiting intensive treatment: a mixed-methods study of preliminary outcomes and acceptability

**DOI:** 10.3389/fpsyg.2026.1843894

**Published:** 2026-07-20

**Authors:** Amber Hulskotte, Katinka Franken, Klaas Huijbregts, Peter M. ten Klooster, Gerben J. Westerhof, Ernst T. Bohlmeijer

**Affiliations:** 1 GGNet, Warnsveld, Netherlands; 2 Department of Psychology, Health & Technology, Faculty of Behavioural, Management and Social Sciences, University of Twente, Enschede, Netherlands

**Keywords:** compassion, group intervention, mental wellbeing, personal recovery, personality disorder, positive psychology

## Abstract

**Introduction:**

A compassion-based positive psychology group intervention (CB PPGI) is a promising approach to supporting personal recovery and mental wellbeing in individuals with severe mental health difficulties.

**Methods:**

This mixed-methods study examined the preliminary outcomes and acceptability of an 8-week CB PPGI in 18 adults with personality disorders (PD) awaiting intensive treatment. Mental wellbeing, psychological distress, and personality functioning were assessed across five time points up to 3 months post-intervention. Qualitative interviews were conducted with participants (*n* = 16, including dropouts) and therapists (*n* = 3) to evaluate acceptability.

**Results:**

Retention at the 3-month follow-up was 67% (12/18 participants). From baseline to follow-up, participation in the CB PPGI was associated with substantial reductions in psychological distress (BSI, *d* = −1.12) and improvements in personality functioning (SIPP-SF, *d* = 0.74). Positive mental health showed a moderate but non-significant increase over time (MHC-SF total score, *d* = 0.50). Emotional wellbeing improved significantly (*d* = 0.60), and psychological wellbeing showed a similar magnitude of improvement (*d* = 0.60), whereas social wellbeing did not (*d* = 0.22). Thematically analyzed qualitative interviews with participants (*n* = 16, including dropouts) and therapists (*n* = 3) indicated that the intervention was generally perceived as acceptable. The group format, strengths-based focus, and therapist support were particularly valued, although participants suggested improvements in pacing, session length, and content.

**Discussion:**

Overall, the CB PPGI appears to be an acceptable and potentially valuable strengths-based intervention for individuals with PD awaiting intensive treatment. However, conclusions regarding effectiveness are limited by the uncontrolled single-arm design. Further controlled studies with larger samples and extended follow-up periods are needed to evaluate effectiveness and broader feasibility.

## Introduction

Personality disorders (PD) represent a major challenge in mental health care due to their pervasive impact on social, occupational, and daily functioning, as well as overall mental wellbeing. They are characterized by enduring patterns of maladaptive cognition, affect regulation, and interpersonal difficulties, which contribute to substantial distress and functional impairments (Bender et al., 2001; Lenzenweger et al., 2007). Although evidence-based psychotherapies—such as schema therapy, dialectical behavior therapy, and mentalization-based treatment—effectively reduce symptom severity (Cristea et al., 2017; Oud et al., 2018), their effects on social participation, occupational functioning, and long-term mental wellbeing remain modest and heterogeneous (Storebø et al., 2020). These limitations underscore the need for approaches that extend beyond symptom reduction to address broader dimensions of recovery.

From a developmental perspective, personality disorders (PD) can be understood as the outcome of disrupted self-development processes that emerge during adolescence. Rather than arising *de novo* in adulthood, personality pathology is thought to reflect earlier disturbances in identity formation and self-functioning (Sharp, 2020). Longitudinal research suggests that impairments in identity integration and self-definition during adolescence are associated with later personality dysfunction (Bogaerts et al., 2023). These disruptions may contribute to enduring difficulties in self-regulation, interpersonal functioning, and identity stability. Emerging evidence further indicates that interventions targeting personality functioning can lead to meaningful improvements, particularly in identity-related domains (Palermo et al., 2026). This developmental perspective is relevant to the present study because it suggests that interventions targeting self-related capacities, such as self-compassion, may address processes central to personality pathology. Self-compassion may be particularly relevant within this framework because it reflects an adaptive form of self-relating that may counteract disruptions in identity formation and self-functioning. Self-compassion involves responding to personal suffering with kindness, non-judgment, and understanding (Neff, 2023) and has been shown to buffer the association between emotional distress and borderline features in adolescents (Carreiras et al., 2022). In addition, self-compassion has been identified as a potential mechanism linking supportive interpersonal experiences to better mental health outcomes (Bai et al., 2025). Together, these findings suggest that interventions targeting self-related processes, including self-compassion, may be relevant for addressing core features of personality dysfunction. This may be particularly relevant for individuals with PD, who often experience elevated shame and self-criticism. Positive Psychology Interventions (PPIs) delivered without sufficient attention to emotional safety may sometimes be experienced as invalidating or unattainable in individuals with severe psychopathology. Integrating compassion-based principles may therefore help create a safer context in which positive psychological resources can be explored.

Individuals with severe mental illness, including PD, often experience chronic distress, long treatment histories, and impairments across multiple life domains (Culina et al., 2024; Leichsenring et al., 2023; Skodol et al., 2002). This highlights the importance of interventions that promote positive mental health. Positive mental health, or mental wellbeing, comprises emotional, psychological, and social wellbeing (Keyes, 2005) and can be defined as the ability to experience joy, meaning, engagement, and relatedness—even in the presence of mental health difficulties (Bohlmeijer and Westerhof, 2020). Lower levels of mental wellbeing and psychological wellbeing in particular, constitute a robust risk factor for the onset and recurrence of mental disorders (Fava and Tomba, 2009; Keyes et al., 2010; Ten Klooster et al., 2025; Wood and Joseph, 2009). Psychological wellbeing closely aligns with the concept of personal recovery (Kraiss et al., 2018; Leamy et al., 2011; Ruan et al., 2025), which is conceptualized as an individualized, self-directed process through which individuals regain agency, identity, and purpose. Personal recovery emphasizes living a meaningful life grounded in personal strengths and values rather than focusing solely on symptom reduction (Anthony, 1993; Leamy et al., 2011; Slade, 2010; Van Weeghel et al., 2019).

The Sustainable Mental Health (SMH) model (Bohlmeijer and Westerhof, 2021) provides an integrative framework for understanding these findings, emphasizing that sustainable recovery requires both the absence of acute symptoms and the presence of sufficient mental wellbeing. Achieving sustainable mental health therefore necessitates targeting both barriers to adaptation (i.e., dysfunctional processes) and resources for adaptation (i.e., functional or positive psychological capacities). PPIs typically aim to cultivate such resources, including strengths awareness and use, gratitude, positive relationships, and optimism (Seligman et al., 2005). Growing evidence indicates that PPIs enhance mental wellbeing and reduce distress in both the general population and clinical groups (Carr et al., 2021; Chakhssi et al., 2018), although research among individuals with severe mental illness remains limited (Geerling et al., 2020; Slade et al., 2016; Valiente et al., 2022).

Compassion can also be considered a positive resource that promotes adaptation and mental wellbeing in the context of mental health difficulties (Gilbert, 2010; Neff, 2023). Compassion has been conceptualized as a fundamental motivational system involving sensitivity to one's own and others' suffering, accompanied by a commitment to alleviate or prevent it (Gilbert, 2014). Self-compassion, the inward flow of compassion, entails responding to personal suffering with kindness, non-judgment, and understanding (Neff, 2023). Self-compassion may be particularly relevant in the context of personality pathology because it reflects an adaptive form of self-relating that may counteract disruptions in identity formation and self-functioning. In adolescent populations, self-compassion has been shown to buffer the association between emotional distress and borderline features (Carreiras et al., 2022) and may function as a mechanism linking supportive interpersonal experiences to better mental health outcomes (Bai et al., 2025). Extensive empirical evidence further demonstrates robust positive associations between self-compassion and mental wellbeing (Ferrari et al., 2019; Finlay-Jones et al., 2023; Kirby, 2025; Zessin et al., 2015). Moreover, self-compassion has been identified as a key mechanism of change in PPIs aimed at enhancing adaptivity and mental wellbeing (Schotanus-Dijkstra et al., 2019; Wang et al., 2025).

Taken together, combining positive psychology and compassion-based interventions may be particularly beneficial for individuals with enduring mental health difficulties. Compassion-based methods promote validation and acceptance of suffering, reducing avoidance and internalized stigma (Gilbert, 2022; Kirby, 2025). This compassionate foundation supports psychological flexibility, enabling individuals to engage with positive experiences—such as joy, meaning, hope, and social connectedness—despite ongoing challenges (Bohlmeijer and Westerhof, 2020, 2021).

Building on these theoretical principles, a transdiagnostic compassion-based positive psychology group intervention (CB-PPGI) has been developed (Kraiss et al., 2023). This 8-week program integrates empirically supported techniques from both compassion-focused and positive psychology traditions. It targets two primary goals: reducing barriers to wellbeing—such as self-criticism, fear of compassion, social isolation, and anxiety—and strengthening adaptive resources, including positive emotions, self-kindness, strengths use, and supportive social relationships. In a large randomized controlled trial, the intervention significantly improved mental wellbeing and personal recovery among individuals with bipolar disorder compared to treatment as usual (Kraiss et al., 2023).

Developed as a transdiagnostic intervention, CB-PPGI may also enhance mental health among individuals with personality disorders. Before conducting intensive, large-scale trials, it is recommended to first conduct a feasibility study (Arain et al., 2010). Feasibility studies serve several methodological functions, including assessing recruitment and retention strategies, evaluating participant and therapist experiences, exploring potential treatment effects, and identifying challenges in implementation (Orsmond and Cohn, 2015). These insights inform the design of future large-scale trials. The present study aimed to evaluate some aspects of feasibility, i.e., the preliminary evidence regarding changes in mental wellbeing, psychological distress, and personality functioning, and the acceptability of the intervention in this clinical population. Acceptability was defined as participants' appraisal of the intervention's appropriateness, engagement, and satisfaction (Proctor et al., 2011).

The study was conducted among individuals on a waiting list for intensive clinical treatment. The intervention may support people with severe mental health difficulties by helping them bridge the waiting period before treatment begins. More importantly, a strengths-based intervention may enhance motivation and self-trust in preparation for clinical treatment. The persistence of mental health difficulties despite long treatment histories may impair agency, positive self-image, and treatment expectancy, while fostering stigma and isolation (Collier and Grant, 2018; Slade, 2010; Van Weeghel et al., 2019). Implementing CB-PPGI prior to intensive clinical treatment may therefore help individuals with PD regain a sense of agency, trust, and positive motivation (DiClemente et al., 2008).

Taken together, the literature on personality functioning, self-compassion, and positive psychology interventions provides a theoretical foundation for the present mixed-methods study. Quantitatively, it was hypothesized that participants would show reductions in psychological distress and improvements in personality functioning and mental wellbeing from baseline to follow-up. Based on previous research on compassion-based and positive psychology interventions, stronger effects were expected for emotional and psychological wellbeing than for social wellbeing.

Qualitatively, it was expected that participants and therapists would generally perceive the intervention as acceptable and meaningful, while also identifying facilitators and barriers related to the group format, experiential exercises, and intervention structure.

## Method

### Study design

This mixed-methods study combined qualitative and quantitative methods to evaluate the acceptability and preliminary outcomes of CB-PPGI. The study primarily focused on acceptability and preliminary clinical changes rather than a full feasibility evaluation. Self-reported questionnaire data were collected at five time points: during initial screening (T1), at the start of the intervention (T2), midway through the intervention at 4 weeks (T3), at the end of the intervention at 8 weeks (T4), and 3 months post-intervention (T5). At the 3-month follow-up assessment (T5), two participants had initiated intensive personality disorder treatment, whereas the remaining participants had not yet started follow-up treatment.

Qualitative data were obtained through semi-structured interviews with both patients and therapists to explore their experiences of the intervention and gather suggestions for improving its content and delivery. These interviews were guided by the Client Change Interview (CCI) framework. The semi-structured interview guide is provided in the Supplementary Table S1.

### Participants

In this study, three therapists and 18 adult patients initiated participation. The patients were allocated to three CB-PPGI groups. Patients were recruited between January and September 2024 from the waiting list for personality-disorder treatment at a specialized mental health center. All participants had a confirmed diagnosis of a personality disorder established in routine clinical practice at the center, based on DSM-5 criteria using routine diagnostic procedures, including structured clinical interviews when indicated and multidisciplinary clinical consensus. Inclusion criteria were: (a) a confirmed diagnosis of a personality disorder, (b) capacity to participate in a group setting, and (c) proficiency in Dutch. Exclusion criteria were acute psychosis and severe substance use disorders. All patients provided written informed consent.

Patients had a mean age of 45.9 years (*SD* = 13.5); 11 identified as female and seven as male. Diagnoses included avoidant, cluster B, cluster C, and mixed cluster B/C personality disorders. Comorbid conditions were common and included trauma-related disorders (33%), mood disorders (28%), ADHD (22%), anxiety disorders (11%), and substance- or behavior-related disorders (22%). Most patients had extensive treatment histories: 56% reported three or more prior treatment episodes, and 50% had been in treatment for 6 years or longer. Demographic and clinical characteristics are presented in Table 1.

**Table 1:** Demographic and clinical characteristics of the CB-PPGI group participating patients (*n* = 18).

Variable	*n*	%	*M*	*SD*
Age	18		45.9	13.5
Gender				
Female	11	61		
Male	7	39		
Relationship status				
Single	4	22		
Married/cohabiting without children	5	28		
Married/cohabiting with children/other	9	50		
Education				
Vocational (MBO)	4	22		
Higher professional (HBO)	5	28		
Other	9	50		
Personality disorder diagnosis				
Borderline PD	1	6		
Avoidant PD	2	11		
Other specified PD, Cluster B	2	11		
Other specified PD, Cluster C	5	28		
Other specified PD, Cluster B/C	8	44		
Comorbid disorders				
Trauma-related disorders	6	33		
Mood disorders	5	28		
ADHD	4	22		
Substance/behavioral disorders	4	22		
Anxiety disorders	2	11		
Eating disorders	1	6		
None	2	11		
Psychotropic and pain medication				
Antidepressants	11	61		
Anxiolytics/benzodiazepines	3	17		
Antipsychotics	3	17		
Stimulants	1	6		
Mood stabilizers	1	6		
Somatic pain medication	2	11		
None	4	22		
Treatment episodes				
1–2	5	28		
3–5	5	28		
6–10	3	17		
>10	4	22		
Other	1	6		
Duration of treatment				
≤ 5 years	4	22		
6–10 years	7	39		
11–20 years	1	6		
>20 years	2	11		
Other	4	22		

### Intervention

The intervention followed the eight-session compassion-based positive psychology group intervention, with a session-by-session outline provided in Supplementary Table S2. The weekly 2-h sessions integrated psychoeducation, experiential exercises, and home assignments, covering themes such as self-compassion, self-soothing, savoring and gratitude, coping with anxiety, personal strengths, and positive relationships. To support self-study between group sessions, patients received the evidence-based self-help book *Using Positive Psychology Every Day: Learning How to Flourish* (Bohlmeijer and Hulsbergen, 2018). Groups consisted of five to seven patients and were facilitated by an experienced psychologist (AH) with expertise in personality disorders and contextual therapeutic approaches, including Acceptance and Commitment Therapy (ACT), supported by a psychology intern. The intervention was delivered using a structured session-by-session protocol and accompanying workbook. Formal fidelity ratings or protocol adherence checks were not conducted.

### Quantitative outcome measures

To assess preliminary changes associated with the intervention, self-reported mental wellbeing, psychological distress, and personality functioning were assessed using validated self-report instruments at five time points (T1–T5). Together, these instruments provided a broad assessment of both psychological distress and positive mental health, consistent with contemporary models of mental health (e.g., Franken et al., 2018; Bohlmeijer and Westerhof, 2021).

The selected outcome measures were chosen to reflect the primary domains targeted by the CB-PPGI. The MHC-SF was included to assess emotional, psychological, and social wellbeing, consistent with the intervention's focus on promoting positive health and adaptive functioning. The BSI-GSI was used to evaluate psychological distress and symptom burden, whereas the SIPP-SF assessed personality functioning, including domains related to self-functioning and interpersonal functioning that are considered central to personality pathology. Although hypothesized mechanisms such as self-compassion and self-criticism were not directly measured, the selected outcomes were intended to capture broader changes theoretically associated with these processes.

#### Positive mental health and wellbeing

The fourteen-item Mental Health Continuum–Short Form (MHC-SF; Keyes, 2002) assesses positive mental health across three dimensions: emotional wellbeing (three items; e.g., happiness, life satisfaction), social wellbeing (six items; e.g., social integration, societal contribution), and psychological wellbeing (five items; e.g., self-acceptance, autonomy, personal growth). The questionnaire consists of 14 items rated on a six-point Likert scale (0 = never to 5 = every day), with higher scores indicating better wellbeing. Both average subscale scores for the wellbeing dimensions and a total average score can be computed with higher scores indicating higher wellbeing and positive mental health. The MHC-SF demonstrates excellent internal consistency (α = 0.89–0.93) and good construct validity (Franken et al., 2018; Lamers et al., 2011). In the current sample, internal consistency for the total scale at baseline (T1) was α = 0.91.

#### Psychological distress

The Brief Symptom Inventory (BSI; De Beurs and Zitman, 2006) is a widely used measure of psychological distress over the past week. It includes 53 items rated on a five-point Likert scale (0 = not at all to 4 = extremely) and yields nine symptom dimensions: somatization, obsessive–compulsive (often translated as cognitive problems in the Dutch version), interpersonal sensitivity, depression, anxiety, hostility, phobic anxiety, paranoid ideation, and psychoticism. The BSI Global Severity Index (BSI-GSI) provides an overall measure of psychological distress and served as the primary distress outcome in this study. Higher scores indicate greater psychological distress. The BSI-GSI shows strong internal consistency (Cronbach's α > 0.90) and good test–retest reliability (*r* = 0.68–0.91) in clinical and general populations (De Beurs and Zitman, 2006). In the current sample, internal consistency of the BSI-GSI at baseline (T1) was α = 0.96.

#### Personality functioning

The Severity Indices of Personality Problems-Short Form (SIPP-SF; Feenstra et al., 2011) assesses core components of personality functioning across five domains: identity integration, relational capacities, responsibility, self-control, and social concordance. The instrument consists of 60 items rated on a four-point Likert scale (1 = not at all to 4 = very much), with higher scores indicating better personality functioning. Subscale scores are computed by averaging the items belonging to each domain, and an overall index of personality functioning can be obtained by averaging all 60 items to create a total score. The SIPP-SF has demonstrated good internal consistency (α = 0.70–0.89) and validity across various clinical populations, including individuals with PD (Verheul et al., 2008). In the current sample, internal consistency of the total scale at baseline (T1) was α = 0.92.

### Qualitative data collection and processing

Acceptability, defined as the degree to which patients and therapists perceived CB-PPGI as appropriate, helpful, and relevant for individuals with PD, was examined through post-intervention semi-structured interviews. To obtain a balanced perspective on acceptability, the views of both patient completers and dropouts, as well those of therapists, were included. The interviews elicited participants' reflections on the content and structure of the intervention and its overall fit with their needs (see Supplementary Table S1). All interviews were conducted by one author (KF), audio-recorded with participants' consent, transcribed verbatim, and anonymized during transcription. To ensure privacy, original audio files were deleted after transcription, and only anonymized transcripts were used for subsequent analyses.

To enhance the trustworthiness of the qualitative analysis, reflexivity was incorporated throughout the analytic process. Two researchers (KF, GW) independently coded a subset of transcripts, after which coding discrepancies and emerging themes were discussed in iterative consensus meetings to refine the coding framework. Consensus was subsequently reached, after which KF recoded all transcripts according to the refined coding framework. In addition, reflexive notes were maintained during analysis to critically examine potential assumptions and interpretive biases, including those related to the intervention's theoretical orientation.

### Data analysis

#### Quantitative analysis

Longitudinal effects of the CB-PPGI intervention were analyzed in IBM SPSS Statistics (Version 28.0.1.1) following an intention-to-treat approach using multilevel linear regression analyses with time (T1–T5) as a repeated effect. This approach accounted for the nesting of repeated measurements within individuals over time and for missing data on the dependent variables using restricted maximum likelihood estimation. All available observations were included in the analyses. The primary outcome was positive mental health (MHC-SF total). Global distress (BSI total) and personality functioning (SIPP-SF total) were defined as secondary outcomes. Subscale scores (MHC-SF and SIPP-SF domains) were pre-specified as exploratory outcomes. For each outcome, time (T1–T5) was first entered as a continuous fixed covariate to test whether outcomes significantly changed over time. Repeated measurements were modeled within participating patients with an unstructured (UN) covariance matrix as this structure provided substantially better fit for the total positive mental health scores (MHC-SF) than a first-order autoregressive or compound symmetry structure according to both the −2 restricted log-likelihood and Akaike's information criterion. Given the small sample size, parameters were estimated with restricted maximum likelihood estimation, and denominator degrees of freedom used the Satterthwaite approximation. Estimated marginal means and standard errors were obtained for each time point by entering time as a fixed factor in a subsequent MLR model. Pairwise comparisons between baseline (T1) and 3 months post-intervention (T5) scores were based on two-sided (unadjusted) least significant difference contrast tests.

For interpretability, we report Cohen's *d* effect sizes as the standardized mean change from T1 to T5, computed from the MLR estimated marginal means and standard errors. Effect sizes > 0.80 were considered large, effects > 0.50 as moderate and effect sizes > 0.20 as small (Cohen, 1988). Positive *d* effect sizes for MHC-SF and SIPP-SF scores indicate increases in mental wellbeing and personality functioning; negative *d* effect size for BSI scores indicates a decrease in symptoms.

#### Qualitative analysis

Thematic analysis, as outlined by (Braun and Clarke 2006, 2019), was used to analyze the interview transcripts. To enhance reliability and reflexivity, two researchers (KF and EB) independently coded two interviews to refine the coding framework, resulting in 100% overlap. The subdivision into sub-themes did not lead to any disagreements between two other researchers (KF and GW). Hence, KF applied the final coding scheme to all interviews and maintained analytic memos throughout the process. The analysis focused on themes related to the perceived acceptability. As the number of interviews was predetermined, thematic saturation was not used to conclude data collection. In total 158 patient and 40 therapist statements were included in the analysis. Themes were iteratively reviewed and refined to reflect patients' perceptions of the intervention and their suggestions for improvement. Thematic analysis thus provided a structured yet flexible framework that complemented the quantitative findings and illuminated patterns in patients' experiences.

### Ethical considerations

The study was approved by the Ethics Committee of University of Twente (reference 230049). All procedures adhered to the Declaration of Helsinki. Participants provided written informed consent and could withdraw at any time. Data were handled in accordance with the GDPR, pseudonymized or anonymized where appropriate, and stored securely within the mental health care center.

## Results

### Participating patient flow and attrition

Twelve patients (six women, six men; *M* = 45.7 years, *SD* = 11.6) completed the program. Six patients discontinued the CB-PPGI. Three patients discontinued due to emotional discomfort but continued to receive usual care while awaiting specialist PD treatment. Among these three, one patient stopped because of caregiving responsibilities, one found the material too complex and subsequently transferred to a service specializing in intellectual disabilities, and one discontinued due to perceived imbalance in facilitator attention among group members. Two patients discontinued for personal reasons unrelated to the intervention (e.g., work–travel constraints). In addition, after three group sessions, one patient continued the intervention individually due to trauma reminders, allowing continued engagement in a more tailored format.

Although 12 participants completed the intervention, questionnaire completion varied slightly across assessment points. At T4, data were available for 11 participants because one completer did not complete the post-intervention assessment. At T5, data were available for 11 participants because another completer did not complete the 3-month follow-up assessment.

### Quantitative results

#### Positive mental health and dimensions of wellbeing

Positive mental health scores across the five measurement points are presented in Table 2. Positive mental health, as measured by the MHC-SF total score, showed a non-significant linear increase between baseline and the 3 months post-intervention follow-up (*p* = 0.135). The mixed-model T5–T1 contrast was ΔM = 0.55 (95% CI [−0.18, 1.27], *p* = 0.129). The standardized mean change from T1 to T5 on the MHC-SF total score suggested a moderate improvement with Cohen's *d* = 0.50.

**Table 2:** Estimated marginal mean total and subscale scores with standard errors of the mental health continuum-short form (MHC-SF) across measurement points.

Time point	Positive mental health	Emotional wellbeing	Psychological wellbeing	Social wellbeing
T1 (*n* = 18)	1.600 (0.187)	1.644 (0.300)	1.890 (0.210)	1.244 (0.191)
T2 (*n* = 13)	1.958 (0.266)	1.968 (0.342)	2.205 (0.315)	1.643 (0.251)
T3 (*n* = 12)	2.078 (0.256)	2.337 (0.272)	2.373 (0.288)	1.575 (0.291)
T4 (*n* = 11)	1.989 (0.266)	2.168 (0.346)	2.449 (0.309)	1.356 (0.286)
T5 (*n* = 11)	2.146 (0.311)	2.445 (0.332)	2.581 (0.318)	1.543 (0.410)
Cohen's *d* (T5–T1)	0.50	0.60	0.60	0.22

At the subscale level (Table 2), emotional and psychological wellbeing showed positive changes over time, although only emotional wellbeing demonstrated a statistically significant T5–T1 contrast. The corresponding T1–T5 standardized mean changes were moderate for emotional [ΔM = 0.80 (95% CI [0.08, 1.52]), *p* = 0.031, *d* = 0.60] and psychological wellbeing [ΔM = 0.69 (95% CI [−0.06, 1.45]), *p* = 0.070, *d* = 0.60] and small for social wellbeing [ΔM = 0.30 (95% CI [−0.59, 1.18]), *p* = 0.480, *d* = 0.22]. This pattern was broadly consistent with the *a priori* expectation that emotional and psychological wellbeing would show stronger changes than social wellbeing.

#### Psychological distress

Psychological distress, measured by the BSI, decreased significantly over time (*p* < 0.001; Table 3). The T5–T1 contrast was ΔM = −0.60 (95% CI [−0.91, −0.28], *p* = 0.001), corresponding to a large standardized mean change (Cohen's *d* = −1.12).

**Table 3:** Estimated marginal mean scores and standard errors of the brief symptom inventory (BSI) total score across measurement points.

Time point	Psychological distress (BSI-GSI)
T1 (*n* = 18)	1.346 (0.147)
T2 (*n* = 13)	1.250 (0.173)
T3 (*n* = 12)	0.961 (0.153)
T4 (*n* = 11)	0.950 (0.212)
T5 (*n* = 11)	0.748 (0.101)
Cohen's *d* (T5–T1)	−1.12

#### Personality functioning

The results of the SIPP-SF suggested a general trend of improvement across all subscales over time (Table 4). Total personality functioning improved significantly over time (*p* = 0.002). The T5–T1 contrast was ΔM = 19.08 (95% CI [5.18, 32.99]), *p* = 0.010, yielding a moderate Cohen's *d* effect size of 0.74. At the domain level (Table 4), effects were largest for identity integration (*d* = 1.04) and self-control (*d* = 0.78), and smaller for relational functioning (*d* = 0.28), social concordance (*d* = 0.34) and responsibility (*d* = 0.23).

**Table 4:** Estimated marginal mean scores and standard errors of the severity indices of personality problems-short form (SIPP-SF) across measurement points.

Time point	Total score	Identity integration	Social concordance	Relational functioning	Self-control	Responsibility
T1 (*n* = 18)	168.19 (6.03)	28.58 (1.48)	36.33 (1.36)	31.72 (2.08)	33.05 (1.84)	38.12 (1.67)
T2 (*n* = 13)	173.51 (7.00)	32.71 (1.87)	36.28 (1.56)	32.47 (2.36)	34.44 (1.93)	38.13 (1.63)
T3 (*n* = 12)	178.67 (6.23)	33.44 (1.85)	36.65 (1.57)	33.66 (2.17)	34.99 (1.89)	39.60 (1.46)
T4 (*n* = 11)	182.14 (6.17)	33.86 (2.34)	36.76 (1.74)	34.81 (1.94)	37.38 (1.77)	39.62 (1.40)
T5 (*n* = 11)	187.27 (6.07)	35.90 (1.83)	38.91 (2.17)	34.22 (2.16)	38.75 (1.60)	39.67 (1.51)
Cohen's *d* (T5–T1)	0.74	1.04	0.34	0.28	0.78	0.23

### Qualitative results

**Figure 1:**
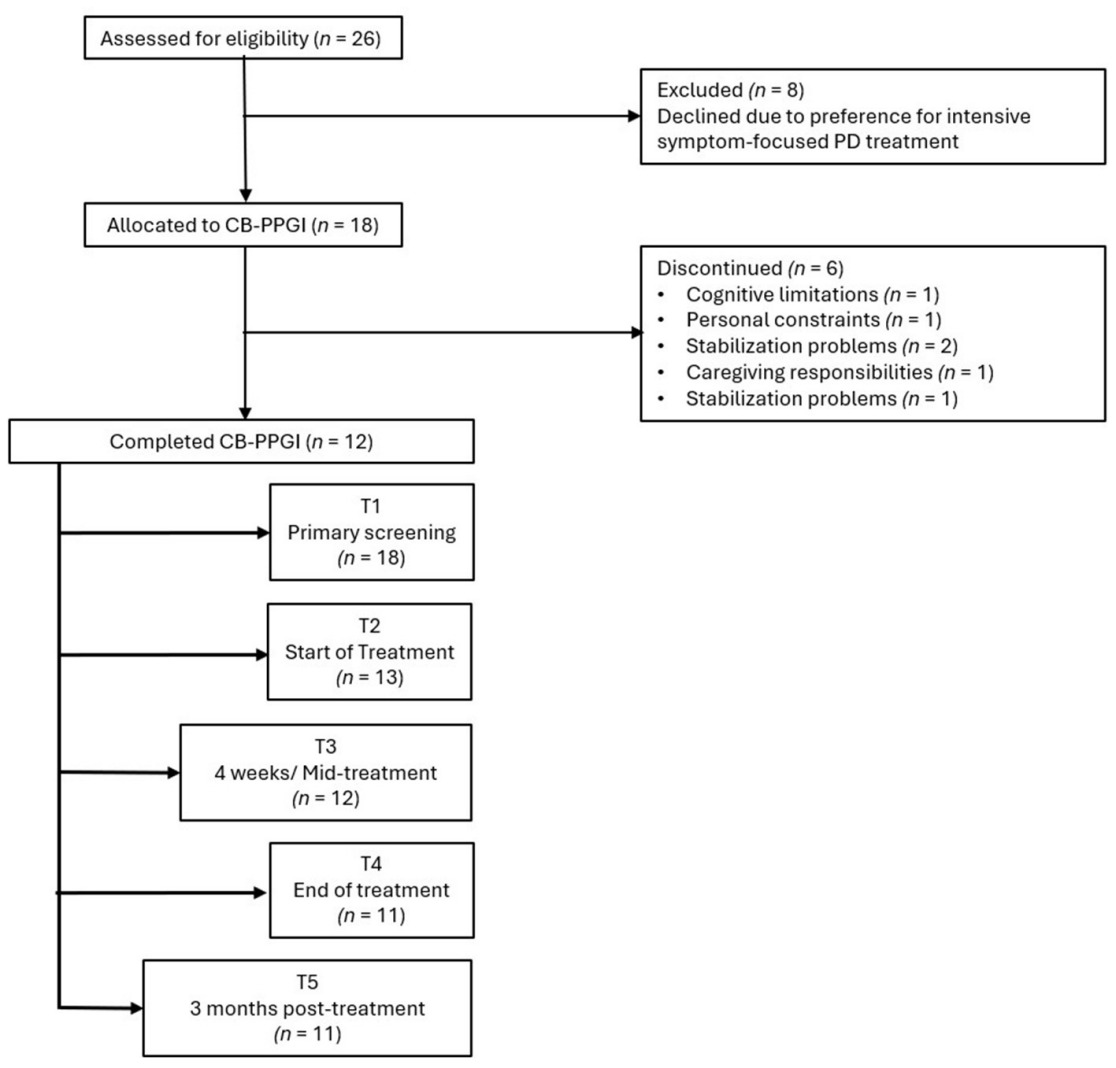
Patient flowchart.

A total of 19s semi-structured interviews were conducted (16 patients; three therapists) to explore patients' experiences with CB-PPGI, including views on program content and structure, as well as suggestions for improving delivery. Table 5 provides a detailed overview of patient themes, subthemes, and illustrative quotes, while Table 6 summarizes therapist perspectives. All 12 patients who completed the eight-session program and four dropouts participated; two early dropouts declined (see Figure 1). Thematic analysis identified broadly overlapping themes across participants. Overall, the CB-PPGI was evaluated positively by the participants; they were particularly pleased with the content and therapist factors. Some dropout participants remained positive regarding various themes, while others reported more mixed experiences and some uncertainty. Key differences included that the completers perceived experiential learning and the relevance of the presented themes as very positive, whereas this was less prominent among dropout participants. Furthermore, completers perceived the positive therapeutic attitude of the therapists more positively than the dropouts (see Supplementary Table S3). Therapists mainly described CB-PPGI as a structured yet flexible approach.

**Table 5:** Themes, subthemes, and illustrative participant quotations.

Theme Subtheme	Illustrative example
Didactics	
Use of theory book and workbook	“The exercises and examples in the theory book were very illuminating. It's written in plain language and easy to read, although I had to reread it occasionally. The chapters aren't very long, which is nice.”
Homework	“I need some motivation. A weekly session helps me keep doing my homework. Then it stays alive and you keep working on it.”
Session duration and length	“I would definitely do it again, complete the entire program. I would become even more mindful. Once a week, but longer, really do homework and feel it, read about the positive aspects, communication, and practice connecting with others during the week. Maybe a second round? I was getting anxious, because it's not over yet.”
Content	
Experiential learning	“I enjoyed the exercises; you had to prepare, practice in the sessions, and then we discussed them again. There were a few times when it stung, but that's okay. The topics discussed were easily applicable to my daily life.”
Relevance of subjects	“They were all meaningful themes that I think were suitable for everyone and could be addressed as needed. These themes really cover a large area. And what I also appreciated that there was a different focus each week.”
Specific exercises	“The exercises ensure that you can think back on them later and sometimes only then realize what insights were given you, such as the four ways of communicating.”
Group aspects	
Group impact	“I was confronted with things I wouldn't normally seek out. And during some exercises, I thought, 'Oh no, I don't know. I can't do that.' Working with others was good for me, and so was seeing other people's reactions. That showed me how it could be okay. If you're completely caught up in emotion, nothing happens. Seeing that was also important to me. And then no one interrogates you completely, and everyone around me reacted understandingly and simply shared my thoughts. That was very helpful.”
Group size	“It was good that the group wasn't too large; a maximum of six ensured that everyone had plenty of opportunity to express their thoughts and feelings.”
Therapist	
Teamwork	“There was good teamwork between the therapists. One was in charge, the other kept track of the time; there weren't two captains on the ship.”
Empathetic listening attitude	“Discussing things in a lighthearted way was helpful for me. You were seen as a person.”
Self-disclosure and equivalence	“The fact that they give a piece of themselves in a group conversation gives us the confidence to show something of ourselves.”
Positive appreciation	“If you want to learn something, you need to be around people who are good at it, not people who are bad at it. It's the same with constantly talking about the negative. Then you stay negative. Then you learn to deal with it in a socially acceptable way. In this training and the way the therapists acted I learned to look at a problem positively.”

**Table 6:** Thematic analysis results on therapists' qualitative descriptions.

Theme	Description	Illustrative examples
Perceived value and impact	The training helped patients shift perspective, build hope, and connect with values. It provided a foundation for future growth.	“Patients learned to see both suffering and beauty.”
Shift toward strengths-based work	The training was seen as a welcome contrast to problem-focused treatment, focusing on resilience, meaning, and growth.	“More about hope and strength than fixing problems.”
Group dynamics and atmosphere	A safe and open atmosphere enabled sharing and growth; peer support played a key role.	“The group felt light and hopeful.”
Therapeutic relationship and role	Therapists' authenticity, compassion, and self-disclosure supported patients; managing group dynamics was the key.	“The therapist brought her heart” “Important to set empathic limits when someone dominates.”
Effective elements of the program	Experiential exercises, metaphors, homework, and psychoeducation were highly valued and impactful.	“Gratitude exercise gave handholds.”
Need for flexibility and depth	Sessions were often experienced as too short; therapists suggested adding time or differentiation (e.g., basic vs. advanced options).	“More time needed per session; Offer optional deepening for those who want it.”

#### Patients' perspectives

Thematic analysis of the patient interviews (*n* = 16) regarding the experience of the CB-PPGI revealed four themes (see Table 5): didactics, content, group aspects, and therapeutic aspects. Table 5 provides an overview of the themes and their descriptions, including illustrative quotes. Patients valued the *didactic elements* of using the theory book alongside the workbook and homework, although some indicated they preferred exclusively in-session learning. Some patients recommended to adjust the pacing and session length while others suggested exploring audio resources such as audiobooks or an app with spoken exercises. With regard to the *content*, patients valued the experiential learning through relevant subjects and specific exercises, which helped them work toward recovery from their mental health issues. Some suggested broadening themes to cover societal issues and physical complaints. Patients also valued the *group* format and size, although one participant preferred to continue individually. With regard to *therapeutic aspects* the positive atmosphere came to the fore that stretched both toward the group and the therapist. This was mentioned in the subthemes of teamwork, empathetic listening attitude, self-disclosure and equivalence, and positive appreciation.

#### Therapist perspectives

Therapists (*n* = 3) converged on the added value and impact of a strengths- and compassion-oriented intervention to enhance motivation and stability awaiting intensive personality-disorder therapy (see Table 6). In the interviews, they highlighted the group dynamics and atmosphere, the supportive relationship and role of therapists, effective elements and depth as well as the need for both flexibility and depth. A suggestion for improvement was the length of sessions.

## Discussion

This mixed-methods study examined changes in mental wellbeing, psychological distress, and personality functioning, and evaluated the acceptability of an 8-week Compassion-Based Positive Psychology Group Intervention (CB-PPGI) for individuals with personality disorders (PD) awaiting intensive day treatment. The findings suggest that CB-PPGI may benefit this population, as the intervention was well received and associated with large reductions in psychological distress, moderate to large improvements in personality functioning, and moderate gains in emotional and psychological wellbeing up to 3 months post-intervention.

### Changes in distress, personality functioning, and mental wellbeing

The findings suggest that individuals with PD may benefit from compassion-based positive psychology while waiting for intensive clinical treatment. Although earlier work demonstrated benefits of the same intervention in individuals with bipolar disorder (Kraiss et al., 2023), personality disorders differ substantially from bipolar disorder in terms of chronic interpersonal difficulties, identity disturbance, shame, and self-criticism. The current findings therefore extend the potential applicability of CB-PPGI to a clinically distinct population characterized by longstanding difficulties in self-functioning and interpersonal functioning. Importantly, the compassion-based components may be particularly relevant for individuals with PD, because they explicitly target shame, self-criticism, and threat-based self-relating processes central to personality pathology (Gilbert, 2022). By combining positive psychology with validation and emotional safeness, the intervention may reduce the risk that strengths-based exercises are experienced as invalidating or overwhelming.

Psychological distress gradually decreased and personality functioning improved, with large effects observed at 3-month follow-up. Emotional wellbeing improved significantly, whereas psychological wellbeing showed more moderate improvements. These results are consistent with growing evidence supporting compassion-based and positive psychology interventions across diverse clinical populations (Carr et al., 2021; Craig et al., 2020; Ferrari et al., 2019; Kirby et al., 2017).

Compassion-based interventions are theorized to improve mental health in individuals with severe or enduring mental illness by enhancing self-compassion, reducing self-criticism, and promoting adaptive regulation of emotions such as shame and guilt (Gilbert, 2022; Kirby, 2025). Self-compassion has been proposed as a psychological resource that fosters safety, connectedness, and acceptance, thereby enhancing resilience and mental health (Finlay-Jones et al., 2023; Gilbert, 2022). The improvements observed in our sample are theoretically consistent with this proposed role of self-compassion as a central mechanism of change in compassion-based interventions; however this could not be empirically tested in the current study. Our findings also indicate that PPIs can play a valuable role in promoting mental health among individuals with severe mental health issues, including PD. By emphasizing strengths, positive emotions, and adaptive coping strategies, PPIs may enhance psychological and emotional wellbeing and reduce distress (Geerling et al., 2020).

From a developmental perspective, the observed improvements in identity integration and self-control are noteworthy, as contemporary models conceptualize personality pathology as rooted in disruptions in self-development and identity functioning (Sharp, 2020). The observed gains in these domains may therefore indicate that the intervention engages processes relevant to personality functioning, which is consistent with emerging evidence that identity-related capacities can improve through intervention (Palermo et al., 2026). However, because self-compassion and related mechanisms were not directly assessed, these interpretations remain theoretical and should be examined in future research.

The early reductions in distress observed in this study are clinically meaningful. Indicators of distress and dysfunction often respond relatively quickly because they reflect proximal targets of intervention, such as emotional stabilization, improved emotion regulation, and restoration of autonomy. These domains are particularly sensitive to common therapeutic factors, including a supportive therapeutic alliance, instillation of hope, and provision of a coherent treatment rationale (Wampold and Imel, 2015). Structured and validating elements emphasized in PD treatments may further contribute to early symptom relief (Bateman and Fonagy, 2015). Early improvements are also prognostically significant: research in broader clinical populations consistently shows that early response predicts more favorable long-term outcomes (Aderka et al., 2012; Haas et al., 2002), and recent machine-learning studies indicate that the absence of early improvement is a strong predictor of later non-response (Franken et al., 2023). These findings suggest that CB-PPGI may help establish a favorable starting point for subsequent intensive treatment.

The moderate gains in emotional and psychological wellbeing are clinically relevant, as psychological wellbeing serves as a buffer against the onset and recurrence of mental disorders (Schotanus-Dijkstra et al., 2017; Ten Klooster et al., 2025; Wood and Joseph, 2009), and positive emotions contribute to mental flexibility and long-term adaptive functioning (Fredrickson, 2001; Fredrickson and Joiner, 2018). The absence of statistically significant changes in overall positive mental health may indicate that an 8-week intervention is insufficient to produce broader changes in eudaimonic and social wellbeing in individuals with longstanding personality pathology. In particular, social wellbeing may require longer periods of behavioral and interpersonal change. It is also possible that social wellbeing may simply not improve as a result of the current intervention, especially since this aspect of wellbeing is not intentionally targeted. Although participants valued the supportive group atmosphere, the intervention primarily emphasized self-compassion, emotional wellbeing, and personal strengths rather than broader social participation. This is consistent with the more pronounced improvements observed in psychological distress and personality functioning, suggesting that symptom-related outcomes may be more proximal and responsive to short-term intervention effects, whereas broader dimensions of wellbeing may follow a slower developmental trajectory. It is therefore plausible that longer follow-up periods, booster sessions, or more intensive or sequential interventions are needed to detect meaningful changes in global wellbeing in this population, as also suggested by evidence from more intensive treatment showing stronger wellbeing gains when these processes are addressed over a longer duration (e.g., Pietersen et al., 2024).

### Integration of quantitative and qualitative findings

The qualitative findings provide important context for interpreting the quantitative outcomes and help clarify how participants experienced the observed changes. Improvements in psychological distress and personality functioning were reflected in participants' appreciation of the experiential learning format, the relevance of the themes addressed, and the practical exercises aimed at recovery and self-understanding. In particular, the focus on strengths, self-kindness, and experiential practice within a supportive group atmosphere was frequently described as meaningful and helpful.

At the same time, the qualitative findings also appeared broadly consistent with the observed improvements in emotional and psychological wellbeing. Participants frequently described increased self-understanding, self-kindness, hope, personal reflection, and a more positive way of relating to themselves and their difficulties, which align with dimensions of psychological wellbeing such as self-acceptance and personal growth. In contrast, changes in broader social wellbeing appeared more limited, suggesting that improvements in social integration and connectedness may require longer periods of practice and support.

Differences between completers and dropouts further underscore the importance of readiness, pacing, and engagement with experiential components in shaping intervention impact. Completers generally described the experiential exercises, thematic relevance, and therapists' compassionate and validating attitudes as highly beneficial, whereas dropout participants more often expressed mixed experiences or doubts regarding these aspects. Together, the quantitative and qualitative findings provide a more integrated understanding of both the potential benefits of CB-PPGI and the contextual factors that may influence engagement and response to the intervention.

### Acceptability and implementation insights

CB-PPGI was generally considered acceptable by both patients and therapists. Participants valued the group format, the explicit focus on strengths and future orientation, and the therapists' compassionate approach, which enhanced engagement. Feedback suggested potential refinements, including adjustments to pacing and session length, broadening thematic content, and providing practical tools for between-session practice and follow-up. These insights indicate that CB-PPGI fits well within contemporary PD care trajectories and offer concrete directions for optimizing delivery.

Six of the 16 participants discontinued the intervention, a rate comparable to other PD-focused treatments (Barnicot et al., 2011; Berghuis et al., 2021; McMurran et al., 2010). In three cases, discontinuation followed emotionally challenging experiential exercises, underscoring the importance of screening for motivation and readiness, pacing sessions carefully, providing stepwise support during experiential components, and offering flexible delivery options (e.g., individual continuation) to sustain engagement.

The current findings tentatively suggest that CB-PPGI may offer meaningful benefits for individuals with a long history of treatment who are awaiting intensive clinical care. By emphasizing personal strengths and resources, the intervention may enhance motivation, agency, and trust in one's capacity for change. In line with the Sustainable Mental Health model (Bohlmeijer and Westerhof, 2021), treatments often first target symptom reduction before implementing interventions that promote resources and mental wellbeing (e.g., Radstaak et al., 2020). However, for individuals with complex and long-standing mental health challenges and extensive treatment histories, offering resource-oriented interventions before renewed clinical treatment may provide a crucial bridge, reinforcing meaning, positive self-images, and a sense of common humanity. This approach aligns with principles of personal recovery (Leamy et al., 2011; Van Weeghel et al., 2019) and may foster renewed motivation, agency, and positive expectations for further clinical treatment. Further research is warranted to examine these potential consequences.

### Strengths and limitations

Strengths of the study include the five measurement points, the mixed-methods design, and the inclusion of dropouts in qualitative interviews. In addition, acceptability was systematically assessed. However, other core feasibility indicators, such as recruitment efficiency, protocol adherence, and implementation fidelity, were not quantitatively evaluated, which limits conclusions regarding overall feasibility.

Important limitations result from the characteristics of an early-stage mixed-methods study and include the small sample size and single-arm design. The absence of a control group precludes causal inference and limits the ability to distinguish intervention-related changes from natural symptom fluctuation, regression to the mean, spontaneous improvement, or other non-specific therapeutic factors. In addition, expectancy effects and common therapeutic factors, such as therapist attention, group cohesion, support, and the provision of a credible treatment rationale, may also have contributed to the observed improvements. A further limitation concerns the diagnostic heterogeneity of the sample. Participants presented with different personality disorder profiles, which may have varied in baseline severity, treatment responsiveness, and trajectories of change. This heterogeneity may have influenced both quantitative outcomes and qualitative experiences of the intervention. A critical limitation of the present study is that hypothesized mechanisms of change, particularly self-compassion and related self-relating processes, were not assessed. As a result, no mediation or process analyses could be conducted, and it is not possible to determine whether the observed changes in outcomes were driven by the theorized intervention mechanisms. Additionally, potential mediating processes—such as self-compassion, adaptability, and positive skills—were not measured, limiting insight into the mechanisms underlying the observed effects. Finally, the generalizability of the findings is limited. The study was conducted in a relatively small, single-site sample of individuals with personality disorders recruited from a specialized waiting list for intensive treatment, which may restrict the extent to which findings can be generalized to other PD populations, treatment settings, or healthcare systems. Participants were help-seeking and already embedded within specialist care pathways, which may have influenced both engagement and outcomes. Moreover, the mixed-methods design, while appropriate for early-stage evaluation, does not allow for population-level inference. As such, the findings should be interpreted as preliminary and hypothesis-generating rather than broadly generalizable.

In addition, adverse experiences were explored qualitatively in interviews and dropout descriptions, but no standardized adverse-event monitoring procedure was used. Therefore, conclusions regarding tolerability and potential negative effects remain preliminary.

### Conclusion and future directions

Before conducting large-scale and intensive efficacy trials, it is essential to first establish feasibility by further examining the acceptability, recruitment potential, and preliminary effect sizes of the intervention (Arain et al., 2010; Orsmond and Cohn, 2015). The moderate to large improvements observed in distress, personality functioning, and mental wellbeing provide valuable guidance for designing future large-scale trials. Combined with the intervention's positive reception, its transdiagnostic applicability, and the practical insights gained regarding delivery, these findings offer a strong foundation for further development and rigorous evaluation. Together with prior work (Kraiss et al., 2023), the present results suggest that CB-PPGI is acceptable and shows preliminary promise for individuals with complex personality pathology awaiting intensive treatment. However, comprehensive feasibility, including recruitment, adherence, and implementation fidelity, remains to be established.

Future research should prioritize randomized controlled trials with active comparators, extend follow-up periods to assess the durability and trajectory of wellbeing, and systematically evaluate implementation outcomes—including reach, fidelity, engagement, tolerability, and criteria for individual continuation. Future trials should also incorporate validated process measures, particularly self-compassion, self-criticism, psychological flexibility, and related self-regulatory constructs, to enable formal testing of the proposed mechanisms of change underlying CB-PPGI. This will allow for mediation analyses and a more precise evaluation of how and for whom the intervention is effective.

## Data Availability

The raw data supporting the conclusions of this article will be made available by the authors, without undue reservation.
