# Supplementary material for: Compassion-based positive psychology group intervention (CB-PPGI) for individuals with personality disorders awaiting intensive treatment: a mixed-methods study of preliminary outcomes and acceptability
Source: Front Psychol. 2026 Jul 20;17:1843894. doi: 10.3389/fpsyg.2026.1843894 (PMC13436829; doi:10.3389/fpsyg.2026.1843894)
Supplement: Supplementary file 1 [file Table_1.docx]

**Table S1.**

*Overview of the Compassion-Based Positive Psychology Group Intervention (*CB-PPGI*) intervention sessions*

| **Session** | **Contents** | **Example (home) exercises** |
| --- | --- | --- |
| **1. Introduction & compassion** | Participants are welcomed and become familiar with each other | Wish yourself something good: be mindful and identify needs and use your inner voice to repeat your compassionate wish. |
|  | Psychoeducation about personal recovery and compassion | Common humanity: realize that negative feelings and experiences are universal. |
|  | Collective compassion exercise |  |
|  | Homework: Fill in handout about personal goals and optimism |  |
| **2. Personal goals & optimism** | Based on the handouts, participants talk about personal goals and wishes and specify personal goals in the group | Imagine your best possible self: visualize yourself in a future where everything has turned out in the most optimal way. |
|  | Individually adjusting personal goals | Letter from the future: write yourself a letter from a future perspective. |
|  | Collective optimism exercise |  |
|  | Homework: Working on personal goals, writing a letter from the future |  |
| **3. Positive emotions** | Read out letter from the future | Three good things: think about three good things that went well today and savor these moments. |
|  | Psychoeducation about positive emotions | Expressing gratitude: identify what you are grateful for in the context of your illness and share those experiences. |
|  | Collective positive emotions exercises |  |
|  | Homework: Taking a photo of a positive moment or experience, working on personal goals |  |
| **4. Coping with fear of relapse** | Sharing photos of positive experiences and talking about the photos | Learn to tolerate and accept fear as important part of life and learn to regulate positive mood and gain a more open view towards them. |
|  | Talking about participant’s fears, how fear is experienced and internal barriers | Compassionate coping with inner fear: learn to be compassionate towards yourself, your emotions and your negative experiences. |
|  | Psychoeducation about fear and (un)healthy emotion regulation strategies |  |
|  | Collective exercises on how to efficiently cope with fear |  |
|  | Homework: Do something you find exciting, perform personal strengths exercise, completing mid-treatment measurement |  |
| **5. Personal strengths** | Identifying strengths | Identifying strengths: describe an activity you enjoy to someone else and he/she names strengths deriving from this activity. |
|  | Psychoeducation about personal strengths | Top 5 strengths: choose your top 5 strengths that give you energy and pleasure. |
|  | Personal goals and strengths: which strengths can be used to achieve personal goals? |  |
|  | Homework: Keep training with exercises, record possible benefits and barriers while performing the exercises |  |
| **6. Positive relationships** | Participants name skills they gathered in the course of the intervention so far and described one example from the last week | Acts of kindness: performing unexpected acts of kindness for someone else. |
|  | Psychoeducation about positive relationships | Active-constructive responding: respond positively to good news shared by someone else; use active communication skills. |
|  | Participants describe a relationship they want to reinforce positively | Expressing gratitude. |
|  | Collective positive relationship exercises |  |
|  | Homework: Keep training with positive relationship exercises |  |
| **7. Compassion** | Psychoeducation about emotional systems and evolutionary background | Develop a compassionate inner voice: write about a situation in the past week where you showed self-compassion. |
|  | Collective exercises mindfulness and compassion | Grandma exercise: imagine a person you feel comfortable with; concentrate on how it feels to be together with this person and savor this moment. |
|  | Coping with thoughts of inferiority and self-critique |  |
|  | Homework: Writing a response to the letter from the second session; fill in questionnaires for post-measurement |  |
| **8. Conclusion** | Talking about results of the questionnaires and figuring out which aspects are going well and which should receive attention in the next weeks |  |
|  | Read out response to the letter from the second sessions |  |
|  | Participants thank each other |  |
