# Supplementary material for: Compassion-based positive psychology group intervention (CB-PPGI) for individuals with personality disorders awaiting intensive treatment: a mixed-methods study of preliminary outcomes and acceptability
Source: Front Psychol. 2026 Jul 20;17:1843894. doi: 10.3389/fpsyg.2026.1843894 (PMC13436829; doi:10.3389/fpsyg.2026.1843894)
Supplement: Supplementary file 2 [file Table_2.docx]

**Table S2.** *Overview of the participants’ interview questions per component and purpose/focus*

| **Component** | **Purpose / focus** | **Example question / prompts** |
| --- | --- | --- |
| **Introduction** | To create a safe, trusting interview context; clarify purpose and voluntary participation; reduce anxiety and support openness | “Before we start, I’d like to explain the purpose of this interview. We are interested in your experiences with the intervention and what it has meant for you. Your participation is completely voluntary, and everything you share will be treated confidentially. You can skip any question or stop at any time. Do you have any questions before we begin?” |
| **Balanced perspective on acceptability** | To explore both positive and negative experiences, ensuring a nuanced understanding of acceptability (what worked well vs. what was challenging or less helpful) | “When you think back on the intervention, what aspects did you find most helpful or valuable?”“Were there also parts that you found less helpful, difficult, or not relevant for you?”“If you look at it overall, what made it a good or less good fit for you?”“Would you recommend it to others? Why or why not?” |
| **Reflections on content and structure of the intervention** | To understand how participants experienced specific components (e.g., exercises, group format, psychoeducation) and the structure (frequency, length, delivery format) | “How did you experience the content of the sessions (for example the exercises or themes discussed)?”“What did you think about the way the intervention was structured (for example the number of sessions, pace, or group format)?”“Were there specific elements that stood out to you, either positively or negatively?”“Did the structure support or hinder your engagement in any way?” |
| **Overall fit with their needs** | To explore perceived alignment between intervention and personal goals, needs, recovery stage, and daily life context | “To what extent did the intervention fit with what you needed at this point in your life?”“Were your personal goals or expectations addressed by the program?”“In what ways did it match—or not match—your situation or challenges?”“Looking back, what would have made it a better fit for you?” |
