# Supplementary material for: Compassion-based positive psychology group intervention (CB-PPGI) for individuals with personality disorders awaiting intensive treatment: a mixed-methods study of preliminary outcomes and acceptability
Source: Front Psychol. 2026 Jul 20;17:1843894. doi: 10.3389/fpsyg.2026.1843894 (PMC13436829; doi:10.3389/fpsyg.2026.1843894)
Supplement: Supplementary file 3 [file Table_3.docx]

**Table S3.** *Valuation per Subtheme per Subgroup of Completers and Dropouts*

| **Theme/subtheme** | **Completers** | | | **Dropouts** | | |
| --- | --- | --- | --- | --- | --- | --- |
| **Valuation** | - ^a^ | + ^b^ |  | - ^a^ | | + ^b^ |
| **Didactics** |  |  |  |  | |  |
| Use of theory & workbook | 8% | 67% |  | 25% | | 25% |
| Homework | 17% | 58% |  | 25% | | 25% |
| Session duration/length | 8% | 17% |  | | 0% | 25% |
| **Content** |  |  |  | |  |  |
| Experiential learning | 0% | 100% |  | | 0% | 50% |
| Relevance of subjects | 0% | 100% |  | | 0% | 50% |
| Specific exercises | 0% | 100% |  | | 0% | 50% |
| **Group aspects** |  |  |  | |  |  |
| Group impact | 17% | 83% |  | | 25% | 50% |
| Group size | 25% | 25% |  | | 0% | 50% |
| **Therapist** |  |  |  | |  |  |
| Teamwork | 8% | 75% |  | | 0% | 50% |
| Empathetic listening | 0% | 84% |  | | 0% | 50% |
| Self-disclosure/equivalence | 0% | 75% |  | | 0% | 50% |
| Positive appreciation | 0% | 91% |  | | 0% | 50% |

*Note.* Percentages indicate the proportion of completers and dropouts who described each theme negatively ^a^ (-) or positively ^b^ (+) in the interviews. Coding was conducted at the participant level.
